# Regular aspirin use among a sample of American Indians/Alaskan Natives in the Upper Midwest region of the United States

**DOI:** 10.1016/j.pmedr.2023.102571

**Published:** 2023-12-20

**Authors:** Sarah M. Alabsi, Sue Duval, Michael Sundberg, Donovan Williams, Russell V. Luepker, Milton Eder, Jeremy R. Van't Hof

**Affiliations:** a University of Minnesota Medical School, Minneapolis MN, USA; b Cardiovascular Division and Lillehei Heart Institute, Department of Medicine, University of Minnesota Medical School, Minneapolis MN, USA; c Department of Medicine, University of Minnesota Medical School, Minneapolis, MN, USA; d Division of Geriatrics, Palliative and Primary Care, University of Minnesota Medical School, Minneapolis MN, USA; e University of Minnesota School of Public Health, Division of Epidemiology and Public Health, Minneapolis, MN, USA; f Department of Family Medicine and Community Health, University of Minnesota Medical School, Minneapolis, MN, USA

**Keywords:** American Indian, Alaska Native, Underserved population, Aspirin use, Preventive therapy, Provider communication, Patient-provider relationship

## Abstract

Despite high prevalence of cardiovascular disease (CVD) and CVD risk factors among American Indian or Alaska Native adults (AI/AN), there is little information on aspirin use in this population. This survey-based study seeks to understand prevalence of aspirin use in a sample of AI/AN adults in the Upper Midwestern United States. In-person and telephone based surveys were conducted querying self-reported CVD and CVD risk factors, aspirin use, and aspirin related discussion with clinicians. A total of 237 AI/AN participants were included: mean age (SD) was 60.8 (8.4) years; 143 (60 %) were women; 59 (25 %) reported CVD history. CVD risk factors were common particularly smoking (37 %) and diabetes (37 %). Aspirin use was much higher among those with CVD (secondary prevention, 76 %) than those without (primary prevention, 33 %). Primary prevention aspirin use was significantly associated with age and all CVD risk factors in unadjusted analyses. After adjustment for demographics and CVD risk factors, only age (aRR 1.13 per 5 years, 95 % CI 1.02, 1.25) and diabetes (aRR 2.44, 95 % CI 1.52, 3.92) remained significantly associated with aspirin. Regardless of CVD status, a higher proportion of those taking aspirin reported a conversation about aspirin with their doctor compared to those not taking aspirin. Among participants with no CVD, those who had such a conversation were 2.6 times more likely to use aspirin than those who did not have a conversation (aRR 2.64, 95 % CI 1.58, 4.44). The findings of this study emphasize the importance of the patient-provider relationship for preventive therapy.

## 1 Introduction

Cardiovascular disease (CVD) is the leading cause of mortality among American Indians or Alaskan Natives (AI/AN).(Heron, 2021) In the Strong Heart Study, cardiovascular mortality among Midwest region AI/AN groups aged 45 to 64 years old was twice as high compared to the United States average.(Lee et al., 1998) In 2017, CVD prevalence among AI/AN adults was nearly 15 %, higher than all other races or ethnicities.(Tsao et al., 2023, Villarroel et al., 2020) As well, CVD risk factors are common in this population with increasing prevalence over the past 25 years. In recent reports, over 25 % of AI/AN adults smoked cigarettes and over half had metabolic syndrome; diabetes alone was 3 times as likely compared to White adults.(Godfrey et al., 2022, Tsao et al., 2023) As of 2006, 79 % of AI/AN had at least one CVD risk factor and 46 % had two or more.(Jernigan et al., 2010) Aspirin has long been a recommended therapy to prevent recurrent CVD and until recently was advised for individuals with high CVD risk before any cardiovascular event. Despite the high prevalence of CVD and CVD risk factors among AI/AN adults, there is little information on aspirin use in this population. This study aims to address this knowledge gap by evaluating aspirin use among a sample of AI/AN adults living in 5 states in the Upper Midwest region of the United States.

## 2 Methods

### 2.1 Study sample and data collection

A series of surveys were conducted in Minnesota, North Dakota, South Dakota, Iowa, and Wisconsin between 2015 and 2020 as part of the “Ask About Aspirin” project.(Duval et al., 2021) The methodology for this study has been previously described in detail.(Krzyzanowski et al., 2019, Van’t Hof et al., 2019) In brief, men aged 45–79 and women aged 55–79 completed surveys in two settings—in-person surveys administered by community health workers in the Minneapolis-Saint Paul metropolitan area, and telephone surveys in the remainder of the state and surrounding states. Surveys were conducted in 2015, 2017, and 2019–20. A total of 8,342 participants self-reported their race and ethnicity. For this analysis, we included participants from all surveys who identified as “American Indian or Alaskan Native.” The terminology used in our surveys was based on terminology used by the Census Bureau. The study excluded those with missing data for sex (4) resulting in a sample size of 237. All individuals gave verbal informed consent, and in-person participants were offered a $10 gift card. The University of Minnesota Institutional Review Board (IRB) approved this study.

### 2.2 Study variables

Surveys queried participant demographics, CVD history and CVD risk factors, regular aspirin use, and aspirin related discussions with care providers. All data were collected by self-report. Individuals who reported past heart attack, stroke, peripheral artery disease or a revascularization procedure were considered positive for CVD history. When considering aspirin use, they were categorized as “secondary prevention” candidates. Those with no CVD history were considered “primary prevention” candidates. Self-reported regular aspirin use for the prevention of heart attack or stroke served as the primary outcome and was defined as daily or every other day aspirin use. To understand potential motivation for aspirin use, participants were asked about past conversations with a healthcare provider involving aspirin use, and whether this conversation was initiated by the provider or participant.

### 2.3 Statistical analysis

Descriptive analyses report categorical data as n (%) and continuous data as mean (standard deviation, SD). Aspirin use was stratified by sex and CVD history. Poisson regression with robust estimation of error variance was used to assess factors associated with aspirin use stratified by primary and secondary prevention. Analyses were adjusted for age, sex, highest education attained and self-reported history of smoking, hypertension, diabetes, and hyperlipidemia. Stata version 16 was used for all analyses. (StataCorp. 2019. *Stata Statistical Software: Release 16*. College Station, TX: StataCorp LLC).

## 3 Results

A total of 237 AI/AN participants were included in the analysis: mean age (SD) was 60.8 (8.4) years and 143 (60 %) were women. Table 1 describes participant demographics, CVD history and risk factors, aspirin use, and communication with providers regarding aspirin use. One quarter of participants had a history of CVD. CVD risk factors were common; current smoking (37 %) and diabetes (37 %) were particularly pervasive. When stratified by sex, smoking was more common among women compared to men (44 % vs 28 %). The prevalence of other CVD risk factors was similar by sex.

**Table 1:** Study participant demographics, CVD risk factors, CVD history and aspirin use among Native Americans/Alaska Natives in the Upper Midwest region of the United States, 2015–2020.

**Characteristic**				
		**Overall** **n = 237**	**Women** **n = 143**	**Men** **n = 94**
Age (years)		60.8 ± 8.4	60.9 ± 8.3	60.6 ± 8.7
Marital status	Married	57 (24 %)	21 (15 %)	36 (38 %)
	Single	96 (41 %)	63 (44 %)	33 (35 %)
	Separated/divorced	61 (26 %)	41 (29 %)	20 (21 %)
	Widowed	23 (9.7 %)	18 (13 %)	5 (5.3 %)
Education	Some high school	47 (20 %)	30 (21 %)	17 (18 %)
	High school graduate	60 (26 %)	34 (24 %)	26 (28 %)
	Some college	84 (36 %)	57 (40 %)	27 (29 %)
	College degree/graduate school	44 (19 %)	21 (15 %)	23 (25 %)
CVD risk factors	Current smoking	88 (37 %)	62 (44 %)	26 (28 %)
	Hypertension	148 (62 %)	87 (61 %)	61 (65 %)
	Hyperlipidemia	116 (50 %)	67 (48 %)	49 (53 %)
	Diabetes mellitus	88 (37 %)	55 (39 %)	33 (35 %)
CVD history	Total	59 (25 %)	31 (22 %)	28 (30 %)
	Myocardial infarction	32 (14 %)	14 (9.8 %)	18 (19 %)
	Stroke	20 (8.4 %)	13 (9.1 %)	7 (7.5 %)
	Peripheral artery disease	22 (9.3 %)	13 (9.1 %)	9 (9.6 %)
	Vascular procedure	20 (8.5 %)	10 (7.0 %)	10 (11 %)
Regular aspirin use		103 (43 %)	52 (36 %)	51 (54 %)
Primary prevention*		58 (33 %)	31 (28 %)	27 (41 %)
Secondary prevention*		45 (76 %)	21 (68 %)	24 (86 %)

This table presents characteristics of 237 study participants who self-identified as American Indian or Alaskan Native on surveys conducted in the Upper Midwest region of the United States from 2012 to 2020. Abbreviations: CVD indicates cardiovascular disease.

*Total number of participants in primary and secondary prevention groups is 178 and 59, respectively.

Aspirin use was much higher among those with CVD (76 %) than those without (33 %, Table 1). Aspirin use was more common among men with and without CVD (24/28, 86 % and 22/66, 41 % respectively) than women (21/31, 68 % and 31/112, 28 % respectively). Regardless of CVD status, a higher proportion of those taking aspirin reported a conversation about aspirin with their doctor compared to those not taking aspirin. Among participants with CVD, conversations were reported by 82 % (37/45) and 64 % (9/14) of those taking and not taking aspirin, respectively. Among individuals without CVD, conversations were reported by 74 % (43/58) and 31 % (37/120) of those taking and not taking aspirin, respectively.

In the primary prevention group (no CVD history), aspirin use was positively associated with older age, hypertension, diabetes, and hyperlipidemia (Table 2) in the unadjusted analysis, but only the association with age (aRR 1.13, 95 % CI 1.02, 1.25 for every 5 years) and diabetes (aRR 2.44, 95 % CI 1.52, 3.92) remained statistically significant after adjustment for age, sex, education, and CVD risk factors. In this primary prevention group, 54 % had a conversation about aspirin with a doctor (Table 2). Participants who had such a conversation were 2.6 times more likely to use aspirin than those who did not have a conversation (aRR 2.64, 95 % CI 1.58, 4.44, Table 2). The secondary prevention group was small (59) and underpowered for regression analyses evaluating aspirin use resulting in wide confidence intervals and no statistically significant association with age, sex, or CVD risk factors.

**Table 2:** Factors associated with primary prevention aspirin use among American Indian or Alaskan Native adults in the Upper Midwest region of the United States, 2015–2020.

		**Aspirin use** **(%)**	**Unadjusted** **Risk Ratio (95 % CI)**	**Adjusted** **Risk Ratio (95 % CI)**
Age (per 5 years)			1.22 (1.08, 1.38)	1.13 (1.02, 1.25)
Sex	Male	41 %	1.48 (0.97, 2.24)	1.47 (0.98, 2.20)
	Female	28 %	Ref	Ref
Hypertension	Yes	42 %	2.01 (1.24, 3.27)	1.29 (0.81, 2.06)
	No	21 %	Ref	Ref
Diabetes	Yes	58 %	3.04 (1.97, 4.68)	2.44 (1.52, 3.92)
	No	19 %	Ref	Ref
Hyperlipidemia	Yes	46 %	1.98 (1.29, 3.05)	1.42 (0.92, 2.21)
	No	23 %	Ref	Ref
Current smoking	Yes	22 %	0.55 (0.33, 0.92)	0.72 (0.42, 1.26)
	No	39 %	Ref	Ref
Any aspirin discussion	Yes	54 %	3.51 (2.11, 5.85)	2.64 (1.58, 4.44)
	No	15 %	Ref	Ref

This table presents the associations of participant factors with aspirin use estimated by Poisson regression with robust estimation of error variance. This analysis included participants with no history of heart attack, stroke, peripheral artery disease or a revascularization procedure – primary prevention candidates – totaling 178. Variables in the adjusted model included age, sex, education, hypertension, diabetes, hyperlipidemia, and current smoking.

## 4 Discussion

This study describes aspirin use among a sample of AI/AN in the Upper Midwest region of the United States from 2015 to 2020. Aspirin use before and after a CVD event was similar to national estimates(Boakye et al., 2021) and conversations with health care providers were positively associated with primary prevention aspirin use.

Primary prevention aspirin use among our study population (33 %) was similar when compared to results from the overall Ask about Aspirin study (31 %)(Luepker et al., 2021), and higher when compared to a nationwide study analyzing aspirin use data from 2019 (28 %).(Boakye et al., 2021).

Although recommendations for the use of aspirin for primary prevention have narrowed recently, it remains a recommended medication for patients after a CVD event and may be considered for those at high risk for CVD.(Davidson et al., 2022, Lawton et al., 2022) This is an important consideration as AI/AN adults have high CVD risk already at a young age when bleeding risk is low.(Breathett et al., 2020) Moreover, common atherosclerotic CVD risk calculators may be insufficient when calculating risk for AI/AN populations. The Strong Heart Study developed a risk calculator for AI/AN adults that included proteinuria in the risk calculation, a variable not used in other risk calculators (Lee et al., 2006).

Consistent with prior studies, CVD risk factors were high in our sample population (Jernigan et al., 2010). Hypertension was the most common CVD risk factor at 63 % and 37 % had diabetes. While current smoking status was the least common, it was well above the national average at 37 %, similar to findings in the Strong Heart Study (Zhang et al., 2015).

Research regarding aspirin use for CVD secondary prevention in the AI/AN population is scarce. A 2014 CVD risk reduction study using a case management intervention included increased aspirin use as a secondary outcome. The intervention contributed to a significant increase in aspirin (and other antiplatelet therapy) prescriptions from 79.5 % to 88.1 %.(Moore et al., 2014) Our study showed a similarly high prevalence of secondary prevention aspirin use. However, given the morbidity and contributing mortality of CVD in this population, the lack of research depicting aspirin usage post-CVD diagnosis is sobering, and more research should be done to evaluate the efficacy and side effects of aspirin among AI/AN.

Finally, our data showed participants who had a conversation with a provider regarding aspirin usage were 2.6 times more likely to report regular aspirin use compared to those who did not, demonstrating usefulness of health communication on an individual level. This positive association was also seen in a similar study with African American Minnesotans (Van’t Hof et al., 2019). There are several ongoing initiatives to reduced CVD risk in AI/AN populations.(Breathett et al., 2020) Considering historical, political, and socioeconomic health impacts for many AI/AN people, community based interventions and/or provider knowledge of these impacts locally are encouraged. While primary prevention aspirin may not be the most important factor, this study supports the positive impact of shared decision making between physicians and AI/AN patients on the use of a CVD prevention medication.

Strengths of our study include CVD risk and aspirin use data from a relatively large sample of AI/AN. Although CVD is high among individuals who identify as AI/AN, as a group they are underrepresented in medical research, and this collection of data on their health behaviors is a step towards improving knowledge and healthcare delivery for this group. There are several limitations to consider. We recognize that the title of “American Indian or Alaska Native” is itself a generalization, failing to account for regional differences in bands, tribes, rural, urban, reservation, care delivery from Indian Health Service (IHS) or non IHS clinical settings. The study was also a non-random sample which may not be representative of this population. Although survey responses were collected over several years and the proportion of AI/AN respondents reflected the proportion of AI/AN residents of the region, the sample size was small, particularly within the secondary prevention group. Lastly, self-reported surveys carry inherent bias, but we have shown validity of aspirin use assessment using this telephone survey (Zantek et al., 2014).

## 5 Conclusion

This study provides recent data on aspirin use for the prevention of CVD among a sample of AI/AN adults in the Upper Midwest region of the United States, a population with elevated CVD risk that is poorly represented in clinical studies. Aspirin use for primary prevention of a first CVD event was more likely after a conversation with a healthcare provider emphasizing the importance of patient-provider shared-decision making for preventive therapy.

## CRediT authorship contribution statement

**Sarah M. Alabsi:** Writing – original draft. **Sue Duval:** Conceptualization, Formal analysis, Methodology, Writing – review & editing. **Michael Sundberg:** Writing – review & editing. **Donovan Williams:** Supervision, Writing – review & editing. **Russell V. Luepker:** Conceptualization, Resources, Supervision, Writing – review & editing. **Milton Eder:** Writing – review & editing. **Jeremy R. Van't Hof:** Conceptualization, Data curation, Supervision, Writing – review & editing.

## Declaration of competing interest

The authors declare that they have no known competing financial interests or personal relationships that could have appeared to influence the work reported in this paper.

## Data Availability

Data will be made available on request.
